# [^68^Ga]Ga-interleukin-2 for imaging activated T-lymphocytes: biochemical characterization and phase I study in normal subjects

**DOI:** 10.1007/s00259-025-07430-9

**Published:** 2025-07-01

**Authors:** Alberto Signore, Filippo Galli, Michela Varani, Giuseppe Campagna, Valeria Bentivoglio, Antonella Accardo, Giancarlo Morelli, Chiara Lauri

**Affiliations:** 1 Nuclear Medicine Unit, Department of Medical-Surgical Sciences and of Translational Medicine, Faculty of Medicine and Psychology, “Sapienza” University, Rome, Italy; 2 Department of Pharmacy, University of Napoli “Federico II”, Naples, Italy; 3 Nuclear Medicine Unit, Sant’Andrea University Hospital, Via di Grottarossa 1035, Rome, 00189 Italy

**Keywords:** [^68^Ga]Ga-IL2, PET/CT, T-lymphocytes, Biodistribution, Human study

## Abstract

**Purpose:**

This study aimed to develop a ready-to-use kit for ^68^Ga-labelling of interleukin-2 (IL2) facilitating PET imaging of activated T-lymphocytes.

**Methods:**

Human recombinant IL2 (hrIL2) and Aldesleukin (desIL2), conjugated with two different chelators (NODAGA and THP) were compared. Conjugated-IL2 was stored, freeze-dried at -80 °C and radiolabelled at room temperature. In vitro quality controls (iTLC, HPLC, SDS-PAGE, spectrometry and binding assays on activated T-lymphocytes) and in vivo biodistribution studies in BALB/c mice, were performed. The shelf life of the lyophilized kits was assessed up to 6 months of storage, by iTLC, HPLC and SDS-PAGE. First-in-human study was conducted in 5 volunteers by performing [^68^Ga]Ga-THP-desIL2 PET/CT acquisitions at several time-points, to assess biodistribution and dosimetry.

**Results:**

Mass spectrometry showed that only one molecule of THP or NODAGA is bound to N-terminus of both IL2 proteins. The most efficient conjugation was observed for THP-desIL2. [^68^Ga]Ga-THP-desIL2 showed higher labelling yield (LY) (59.13 ± 2.58%), radiochemical purity (RCP) (97.91 ± 0.45%) and binding affinity to its receptor on activated T-cells (Kd = 0.584 nM/L) and a more favorable biodistribution in pre-clinical studies, with rapid kidney metabolism and lower liver uptake than [^68^Ga]Ga-NODAGA-desIL2 and [^68^Ga]Ga-THP-hrIL2. Lyophilized kit of THP-desIL2 remained stable at -80 °C up to 6 months maintaining high RCP and LY. Phase I study showed a rapid plasma clearance, renal metabolism, safety and favorable dosimetry.

**Conclusions:**

We developed an efficient lyophilized kit of THP-desIL2 for ^68^Ga-labelling at room temperature in GMP conditions, obtaining excellent in vitro and in vivo results. [^68^Ga]Ga-THP-desIL2 PET/CT studies in humans showed favorable dosimetry and safety, thus highlighting its potential for a wide range of clinical applications, particularly in immune-mediated diseases and cancer.

**Supplementary Information:**

The online version contains supplementary material available at 10.1007/s00259-025-07430-9.

## Introduction

As demonstrated by the presence of inflammatory activated T-cells in biopsies or histologic specimens of many cancers and autoimmune diseases, it is, nowadays, well consolidated that chronic inflammation plays a crucial role in their pathogenesis and evolution [1, 2]. Furthermore, in the past decades, several target immunotherapies have been developed aiming at enhancing immune system activity or suppressing inflammatory signaling pathways in cancers [3] as well as in autoimmune diseases [4].

In this view, molecular imaging is becoming a more and more attractive, allowing to non-invasively assessing immune-cell trafficking for early diagnosis, patient selection to specific treatments and therapy follow-up [5–7]. Amongst the plethora of molecules that are currently under investigation [8], interleukin-2 (IL2) has already demonstrated, in the past decades, the great potential to evaluate activated T-lymphocytes in chronic inflammatory diseases, autoimmune disorders and cancer, thus offering the unique opportunity to non-invasively assess disease activity and to monitor therapeutic efficacy [9].

IL2 is a small cytokine (15-KDa) produced by CD4 + T-cells that stimulates lymphocyte proliferation, growth and differentiation [10]. IL2 receptor (IL2R) is composed by three chains, CD122, CD132 and CD25 that confers the ability to bind IL2 with very high affinity to its own receptor. IL2R is mainly expressed on activated T-lymphocytes, in particular on immunosuppressive regulatory T-cells (T-regs), and to a lesser extent on effector cytotoxic T-cells (CD8 +), and natural cells (NKs) [11]. When several stimuli activate T-cells, they start overexpressing the CD25 subunit of IL2R [11]. Therefore, CD25 is considered the most specific marker of T-cell activation and CD25 + cells have shown to have a specific role in TME and in many autoimmune and chronic inflammatory diseases. Thus, imaging CD25 + activated T-cells has become an attractive goal for molecular nuclear medicine.

Native human recombinant protein (hrIL2) became available at the end of ‘80 s but was soon replaced by recombinant aldesleukin-2 (desIL2) as immunotherapy (Proleukin®, Novartis) for metastatic melanoma and renal cell carcinoma [9, 10, 12]. As compared to hrIL2, desIL2 has a lower molecular weight (MW) (15415 Da vs 15330 Da) due to substitution of Cysteine-125 with a Serine residue and the absence of Alanine-1, to improve stability and plasma half-life.

Pre-clinical and clinical studies using radiolabelled IL2, for both single photon emission tomography (SPECT) and positron emission tomography (PET), have demonstrated its great potential and versatility in detecting, quantifying and monitoring activated T-lymphocytes in a wide range of diseases [13–27]. The use of Iodine-123, ([^123^I]I-hrIL2), provided excellent in vitro and in vivo results [13–17] but was too expensive to be used routinely. Technetium-99metastable ([^99m^Tc]Tc) was adopted (with either MAG3 or HYNIC as chelators) obtaining excellent results in terms of bio-distribution, toxicity, dosimetry, target/background ratio and specific targeting ability to CD25 + cells in different autoimmune diseases and in melanoma patients [18–24]. These clinical studies clearly highlighted the great potential of labelled-IL2 in selecting patients candidate to specific immunotherapies, monitoring their efficacy and predicting prognosis. In particular, in melanoma patients, after demonstrating the correlation between [^99m^Tc]Tc-HYNIC-IL2 and CD25 + cells detected at immunohistochemistry within TME [19], this strategy was also successfully used to predict response of each single lesion to Ipilimumab therapy in metastatic patients [24]. Nevertheless, labelling procedure was time-consuming (> 4 h), thus limiting its routine use. The labelling of IL2 has then been attempted by using Fluorine-18 ([^18^F]F) [25–31], aiming at obtaining higher image resolution. Nevertheless, the labelling procedure is complex, expensive and the images showed very high background activity. Compared to [^18^F]F-IL2, [^68^Ga]Ga-IL2 offers several advantages: lower cost, faster labelling and shorter half-life that could perfectly fit with the short biologic half-life of IL2. However, the choice of the chelating agent is crucial to preserve the structure and function of IL2 and to possibly allow the labelling with Gallium-68 at room temperature, thus avoiding denaturation of IL2. N-(2-Acetamido)iminodiacetic acid (NODAGA) has already demonstrated to be feasible for labelling IL2 with Gallium-68 and it has been recently compared with a fluorinated-IL2, achieving good in vitro and in vivo results [28].

The aim of present study was to set-up a cheap, efficient and reproducible method to radiolabel IL2 with Gallium-68, at room temperature. The underlying hypothesis is that [^68^Ga]Ga-IL2 can preserve its binding affinity to activated T-lymphocytes, confirming the previous findings observed with other SPECT and PET radioisotopes.

We compared two different IL2 molecules (desIL2 and hrIL2) conjugated with two different chelating agents, namely NODAGA and maleimide tris-hydroxypyridinone (THP) and we assessed several quality controls (QCs), biodistribution in BALB-c mice, phase I study and dosimetry in humans.

## Materials and methods

### Biochemical characterization of different batches of IL2 conjugated with different chelating agents

Four different experimental batches of IL2, for labelling with Gallium-68 at room temperature, were compared.

- Formula 1: hrIL2 conjugated with NODAGA-NHS (NODAGA-hrIL2);
- Formula 2: hrIL2 conjugated with Mal-THP (THP-hrIL2).
- Formula 3: desIL2 conjugated with NODAGA-NHS (NODAGA-desIL2);
- Formula 4: desIL2 conjugated with Mal-THP (THP-desIL2);

Proleukin (Aldesleukin, Novartis, desIL2) is commercially available in lyophilized form. Human interleukin (hrIL2) was purchased by ProSpec (code CYT 209, ProSpec, Rehovot, Israel) in lyophilized form. Both desIL2 and hrIL2 were chemically modified using bifunctional chelating agents (THP or NODAGA), commercially available from CheMatech (Dijon, France).

Experiments performed with the above-mentioned formulations are summarized in Table 1.

**Table 1:** Summary of experiments performed with the four IL2 formulations

Formula	Mass spectrometry	Cell binding assay	Biodistribution in BALB/c mice	Kit formulation and QCs	Phase I human study
NODAGA-hrIL2	√	-	-	-	-
THP-hrIL2	√	√	√	-	-
NODAGA-desIL2	√	√	√	-	-
THP-desIL2	√	√	√	√	√

*QC*, quality controls

#### Chemical modification of IL2 proteins with the two chelating agents

Each protein vial was reconstituted in 500 µL of water, and then divided into two aliquots (250 µL), each one used for dissolving an amount of chelating agent powders. The bifunctional chelating agents were analytically weighed in order to guarantee 40-fold excess in final reaction condition.

For the conjugation with THP, 250 µL of reconstituted protein solutions was added to 250 µL of phosphate buffer solution 0.100 mol/L at pH = 8.3 to dissolve 1.32 mg of THP. Sonication was performed using a digital ultrasonic bath (ARGOLab DU-32S) for 20 s at 40% amplitude to ensure complete dissolution and obtain a clear solution. The pH was checked after the dissolution and remained stable (7.2 < pH < 7.8).

For the conjugation with NODAGA, 250 µL of reconstituted protein solution was added to 250 µL of deionized water to dissolve 1.05 mg of NODAGA, in order to reconstitute the original conditions obtained during protein lyophilization (pH 5).

In all the four formula, limpid solutions were obtained, without modification of the final pH value after the powder dissolution. All samples were mechanically mixed using an IKA RW20 overhead stirrer at 500 rpm for 4 h at room temperature. Then, samples were dialyzed for 24 h using using Pur-A-Lyzer™ Maxi Dialysis Kit (3.5 kDa MWCO) against a dialysis buffer containing 0.271 mol/L glycerol, 0.64 mmol/L sodium dodecyl sulphate, 1.41 mmol/L NaH_2_PO_4_ and 6.27 mmol/L Na_2_HPO_4_. At the end of the dialysis, all samples were freeze-dried and lyophilized.

#### Protein digestion procedure

Protein samples, suspended in 50 mmol/L ammonium hydrogen carbonate, were digested for 16 h at 37 °C using trypsin. The obtained peptide blends were purified using a C18 zip-tip (Merk Life Science S.r.l., Milano, Italy), dried and re-suspended in water containing 0.2% trifluoroacetic acid.

#### Mass analysis

Mass analysis of pure, modified and digested samples was performed at CEINGE Laboratory (Naples, Italy). Lyophilized powders of pure and modified proteins were suspended in 50 µL of a mixture containing CH_3_CN/H_2_O (0.2% HCOOH) at 1/1 volumetric ratio. Samples were analyzed by direct injection (flow = 10 µL/min) using ESI–MS Q-TOF Premier (Waters, Milford, MA, USA). Raw data were analyzed using MassLynx 4.1 (Waters, Milford, MA, USA) software.

#### Matrix-assisted laser desorption/ionization mass spectrometry (MALDI-MS) analyses of peptide mixture

Matrix-assisted laser desorption/ionization mass spectrometry (MALDI-MS) analyses were carried out on a 4800 plus MALDI TOF-TOF mass spectrometer (AB Sciex) equipped with a reflectron analyzer and used in delayed extraction mode with 4000 Series Explorer v3.5 software. For the analyses, 0.50 µL of peptide mixture were mixed with an equal volume of α-cyano-4-hydroxycynnamic acid as matrix (10 mg/mL of powder in H_2_O 0.2% TFA in 70% CH_3_CN *v/v*), loaded onto the metallic sample plate and air-dried. MALDI-MS data were acquired over a mass range of 400–5600 m/z in the positive-ion reflector mode. Raw data were elaborated using the software provided by the manufacturer.

#### Identification of peptides by Liquid Chromatography Mass Spectrometry (LC–MS/MS) analysis

Peptide mixtures were re-suspended in 0.2% HCOOH and analyzed by Liquid Chromatography Mass Spectrometry (LC–MS/MS), using a 6530 QTOF LC/MS (Agilent, Santa Clara, CA, USA) system equipped with a nano-HPLC. After loading, peptide mixtures were first concentrated and desalted on the pre-column. For proteins identification, the raw data obtained from the LC–MS/MS analysis were used to search both proteins databases by an in-house version of the Mascot software. The selected parameters for peptide identification were the following: Fixed Modifications: Carbamidomethyl (C), Variable modification: Oxidation (M), Gln- > pyro-Glu (N-term Q), pyrocarbamidomethyl (N-term C) Max, missed cleavages.

### Radiolabelling and quality controls (QCs)

The labelling of each batch was performed with GAIA synthesis module (Elysia-Raytest®, Belgium) using a Germanum-68/Gallium-68 (Eckert-Ziegler, Berlin, Germany) generator, under GMP condition to obtain a sterile product, with incubation at room temperature for 15 min followed by tC2 purification with EtOH to eliminate unbound Gallium-68.

Briefly, 540 MBq of a [^68^Ga]Ga-Cl_3_ solution eluted from Germanum-68/Gallium-68 generator were transferred to a sterile glass vial containing 50 µg of previously conjugated IL2. After 15 min of incubation at room temperature, a small aliquot was used to assess the labelling efficiency (LE) by instant thin layer chromatography (iTLC).

At the end of the radiolabelling process, we obtained a single dose contained 5 ml [^68^Ga]Ga-IL2 (20–30 μg since approximately 50% of IL2 remains in the tC2 column) in a 0.9% NaCl solution with 10% EtOH (dose 74–111 MBq; pH = 6–8).

The percentage of labelling yield (LY) was automatically calculated by the GAIA software, due to the presence of radio-detectors installed in the unit.

The percentage of radiochemical purity (RCP) was calculated by iTLC using two different mobile phases to distinguish free isotope, colloids and [^68^Ga]IL2:

1. MeOH/NH_4_OAc (1:1), for determining the LY
2. 5% NaCl/MeOH/25% NH_3_ (3:1:1), for determining the presence of colloids.

Negligible amount of free or colloidal Gallium-68 was observed (< 3%) at radio-chromatogram.

For each batch the specific activity (SA), in MBq/µg was calculated by using the following formula:

$$-\text{final activity}\;(\text{MBq})/\text{quantity of protein}\;(\mu\text{g})$$

In addition to iTLC, high-performance liquid chromatography (HPLC) was also performed to separate, identify and quantify all components.

### In-vitro binding to activated T-lymphocytes

#### Isolation of human lymphocytes subsets and phenotypic analysis with fluorescence-activated cell sorter (FACS)

To evaluate the expression of IL2R α-chain (CD25) on human peripheral blood mononuclear cells (hPBMC) we used fluorescence-activated cell sorter (FACS, Becton–Dickinson, Franklin Lakes, New Jersey, USA) analysis.

hPBMC were isolated through density gradient centrifugation from the buffy coats of 4 healthy donors, and stimulated 72 h in culture with phytohemagglutinin-M (PHA-M).

Cells were centrifuged and resuspended in PBS at optimal concentration (1 × 10^7^/mL). The monoclonal antibodies anti-human CD25 (ThermoFisher Scientific, Roma, Italy) were added to the cells and incubated for 20 min at room temperature. Then, a small aliquot of cells was analyzed by FACS.

#### Kinetic cell binding assay

For kinetic binding assay, hPBMC from 3 different healthy donors were activated in culture for 3 days in presence of PHA-M (5 mg/mL) and then plated in a Petri-dish suitable for LigandTracer (Uppsala, Sweden) as described elsewhere [32, 33].

To allow the attachment of the suspended cells to the Petri-dish, the surface of glass-bottomed culture dish was treated aseptically with 4 mL of BAM (Sunbright^®^ OE-040CS, NOF Corporation, New York, USA) (2 mg/mL in Milli-Q® water) and incubated for 30 min at room temperature [33]. After washing, 4 mL of cells (2.5 × 10^6^/mL) were plated, left for 40 min at room temperature, then washed with PBS to remove cells not immobilized. Radiolabelled IL2 was added to the cells and counting started for 60 min. After the initial 60 min of association phase, the medium containing un-bound radioligand was gently replaced with fresh PBS, initiating the dissociation phase. The kinetic of cell binding over time, as well as the dissociation from cells, was evaluated for three of the four IL2 formulations. The drop in signal observed after 60 min corresponds to the beginning of the dissociation phase, following the replacement of the radioligand-containing solution. Raw counts per minute (cpm) of cells were corrected for background signal and for isotope decay. Fitting of the kinetic binding data was not constrained to zero at the final timepoint, allowing the model to account for residual binding. Three independent experiments were performed for each IL2 formulation.

### Biodistribution studies in mice

Animal experiments were carried out in compliance with the local ethics committee and in agreement with the National rules and the EU regulation (Study 204/2018‐PR). Biodistribution studies were performed with [^68^Ga]Ga-NODAGA-desIL2, [^68^Ga]Ga-THP-hrIL2 and [^68^Ga]Ga-THP-desIL2 in 36 BALB/c mice (8‐week‐old females) purchased from Envigo (Indianapolis, Indiana, USA). Mice were divided in 3 groups of 12 mice each, according with the 3 different batches of IL2 to be tested.

Approximately 3.7 MBq (100 µCi of labelled IL2 in 100 μl) were injected into the tail vein of each animal.

Due to different SA of different IL2 formulations, in order to inject 3.7 MBq (100 µCi), we administered approximately 6.5 µg of [^68^Ga]Ga-NODAGA-desIL2, 0.6 µg of [^68^Ga]Ga-THP-hrIL2 and 0.5 µg of [^68^Ga]Ga-THP-desIL2.

After each time point (15 min, 1 h, 2 h) 4 mice from each group were anaesthetized and sacrificed; blood samples and major organs (bowel, kidneys, spleen, stomach, liver, muscle, bone and lungs) were collected and weighted. Radioactivity was counted in a single-well γ-counter (PerkinElmer, Waltham, MA, USA) and the percentage of injected dose per organ (%ID) and percentage of injected dose per gram (%ID/g) were calculated, as well as the spleen/blood, spleen/muscle and spleen/bone ratios, being the spleen the only organ on which we expect an accumulation of radiolabelled IL2 due to the presence of activated T lymphocytes in physiological conditions.

### Development of a ready-to-use lyophilized kit for labelling with ^68^Ga

As previously mentioned, after conjugation, THP-desIL2 was freeze-dried, lyophilized and stored at -80 °C. Different vials were reconstituted after 1, 2, 3 or 6 months.

Sodium-dodecyl-sulphate poly-acrylamide gel electrophoresis (SDS-PAGE) and HPLC were performed at each time point after the reconstitution of each batch, before radiolabelling.

iTLC and HPLC were also performed after ^68^Ga-labelling to evaluate SA, LY and RCP.

In vitro stability over time was evaluated in human serum at 37 °C. The radiolabelled compound was incubated in 1 ml final solution and an aliquot of 100 µl was analyzed by iTLC after 10 min, 1 h, 2 h, 3 h and 4 h to calculate free Gallium-68 released from IL2.

### Biodistribution and dosimetry of ^68^Ga-THP-desIL2 in humans

Phase-I human study [^68^Ga]Ga-THP-desIL2 was approved by the Ethic Committee of “Sapienza” University of Rome on the 15/05/2020 (SMART CIG: Z4B316CBDF; EudraCt number: 2020–001749-38), and all subjects signed an informed consent form.

After performing all quality controls of [^68^Ga]Ga-THP-desIL2, the mean and standard deviation of the administered mass and activity of [^68^Ga]Ga-THP-desIL2 were 25.4 ± 3.1 µg (range 22–30 µg) and 159.6 ± 36.9 MBq (range 78–206 MBq) (4.31 ± 0.9 mCi), respectively. Whole body PET/CT images were acquired at 15’, 30’, 60’, 90’, 120’, 150’ and 210’ after i.v. administration in 5 healthy male volunteers (age range: 28–45 years) to assess biodistribution and for dosimetric purposes (IDAC-Dose 2.1). THP-desIL2 was previously lyophilized and stored at -80 °C. All subjects were investigated with THP-desIL2 stored for less than 3 months and no adverse events occurred after the administration of the radiopharmaceutical.

Decay corrected whole-body PET/CT scan was acquired with a dedicated hybrid PET/CT Biograph (Siemens, Germany). After a scout CT for the definition of field of study, a low-dose CT scan (120 mA), without contrast, was acquired for attenuation correction.

Both qualitative and semi-quantitative assessment by using maximum and mean standardized uptake value (SUVmax and SUVmean, respectively) were performed, to assess the in vivo biodistribution of [^68^Ga]Ga-THP-desIL2 in each organ.

Semi-automatic organ segmentation was performed to calculate organ volumes and to construct time-activity curves for each organ. Data from all volunteers were pooled; average organ volumes and average time-activity curves were uploaded on IDAC-Dose2.1 software for absorbed dose and effective dose calculation.

### Statistical analysis

The statistical analysis was performed by SAS v.9.4 (SAS Institute Inc., Cary, NC, USA). Continuous variables are shown as mean ± standard deviation (SD) and 95%CI (Confidence Interval). To compare values of LY, SA and RP of lyophilized kits over time, as well as the stability of [^68^Ga]Ga-THP-desIL2 in serum at 37 °C, we used Generalized Linear Mixed Model (GLIMMIX) for repeated measurements. The comparison of binding affinity of different IL2 formulations and of Spleen/Blood ratios in mice was performed by GLIMMIX/GLM procedure, considering Gaussian function as distribution. The normality of distribution of residuals was verified by Shapiro–Wilk test and checking Q-Q plot. Homoscedasticity was verified analyzing the studentized residuals. Post hoc analysis was performed by Tukey’s method. A p-value < 0.05 was considered statistically detectable.

## Results

### Biochemical characterization of different batches of IL2 conjugated with different chelating agents

For both NODAGA and THP we found that a 40:1 chelator:protein ratio was optimal. This ratio was chosen in order to obtain the highest LY and SA with unmodified binding affinity, after testing other several mixing ratios. Ratios below 40:1 (20:1 and 10:1) resulted in a lower labelling efficiency. Ratios above 40:1 (100:1) reduced the binding affinity of labelled IL2 to activated T-cells.

**Fig. 1:**
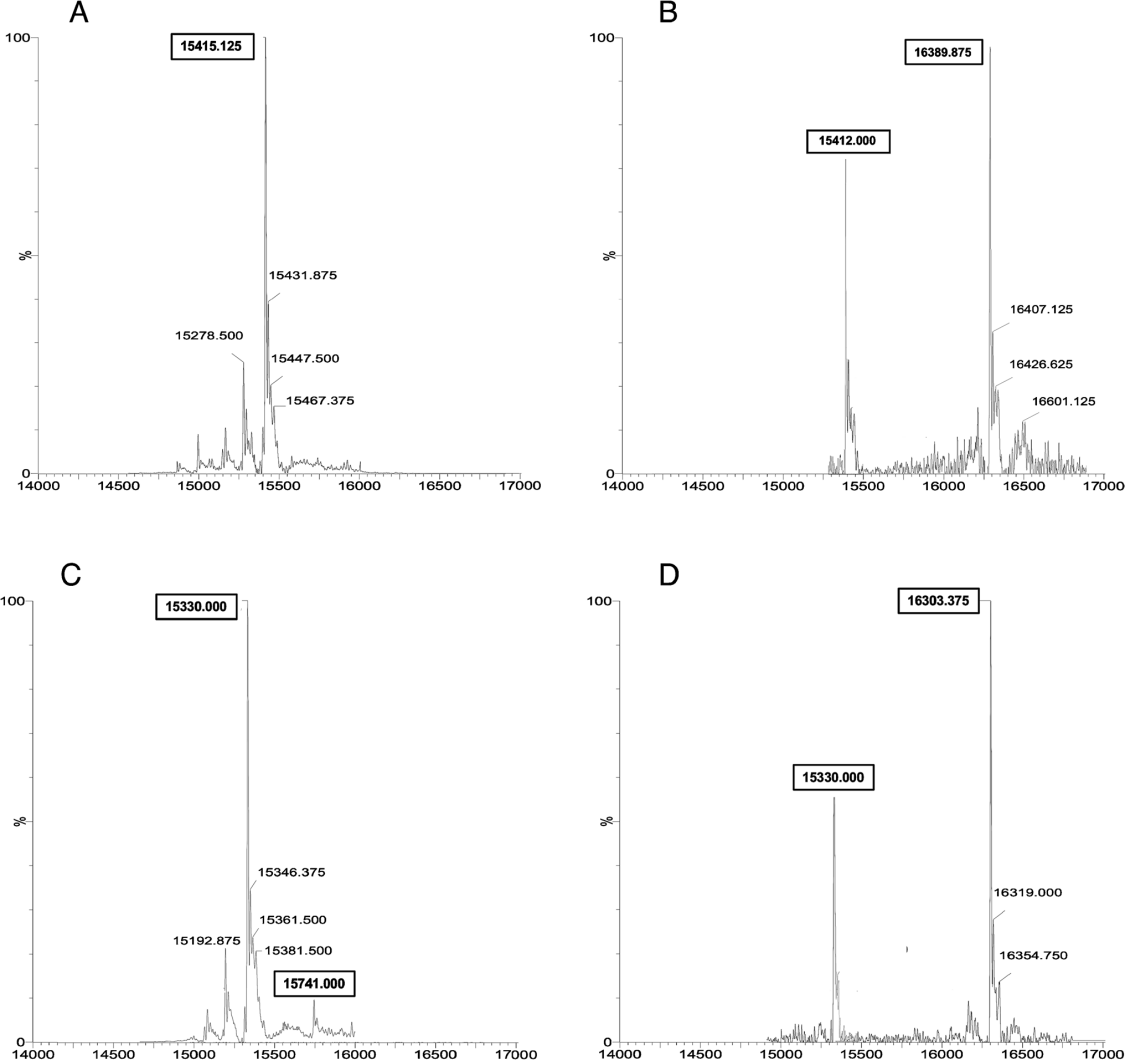
Mass spectrometry of hrIL2 (A and B) and desIL2 (C and D) after the conjugation with NODAGA (A and C) or THP (B and D) chelating agent. Approximately 0.6% of hrIL2 (MW: 15415 Da) is conjugated with NODAGA (**A**), whereas almost 59% of hrIL2 can be chelated with THP (MW: 16389 Da), (**B**). Approximately 5% of desIL2 (MW: 15330 Da) is conjugated with NODAGA (MW: 15741 Da) (**C**), whereas almost 79% of desIL2 is chelated with one molecule of THP (MW: 16303 Da), (**D**) Molecular weight of NODAGA-NHS is 358 Da and of THP-MAL is 919 Da

The mass characterization of the product of the reaction between hrIL2 and NODAGA clearly showed the presence of only the unmodified hrIL2 protein (only 0.6% of IL2 was conjugated), thus indicating that the reaction of the NODAGA on the hrIL2 protein does not happen in our experimental conditions (Fig. 1A). Since no conjugation was observed, NODAGA-hrIL2 was excluded from other experiments.

By comparing the mass spectrometry of the hrIL2 protein, before and after the conjugation with THP, we observed an increase of molecular weight, which corresponds to one THP molecule bound to IL2 with different hydration state of the molecules (Fig. 1B). After digestion of the modified protein, the mass analysis of the peptide pool, by MALDI-TOF and LC–MS/MS allowed to determine the sequence and identification of all peptides, as reported in Table 2. Results showed that THP binds only to the N-terminus of the protein. As expected, THP did not bind to Cys residues, in our experimental conditions at pH = 7.5, since this occurs only in acidic conditions.

**Table 2:** Summary of the mass values identified in the MALDI and LC–MS/MS analysis and assigned to the corresponding peptides in the hrIL2 protein

Sequence position	[MH] + (Dalton)	Aminoacid sequence
1–8	1697.83	APTSSSTK-***THP***
1–8	835.41	APTSSSTK
10–43	4054.26	TQLQLEHLLLDLQMILNGINNYKNPKLTRMLTFK
33–38	728.42	NPKLTR
36–43	1009.44	LTRMLTFK
39–43	639.34	MLTFK
44–48	685.46	FYMPK
49–54	689.41	KATELK
56–76	2620.41	LEEVLNLAQSKNFHLRPRDLI
77–81	386.36	SNINV
98–120	2684.27	SETTFMCEYADETATIVEFLNR

*Sequence of hrIL2*

APTSSSTKKT QLQLEHLLLD LQMILNGINN YKNPKLTRML TFKFYMPKKA TELKHLQCLE EELKPLEEVL NLAQSKNFHL RPRDLISNIN VIVLELKGSE TTFMCEYADE TATIVEFLNR WITFCQSIIS TLT

MH + = protonated monoisotopic mass

Similarly to THP-hrIL2, the analysis of THP-desIL2 showed a mass value indicative of protein modification with only one chelating agent, although in both cases we used a 40-fold excess of the chelating agent over the protein (Fig. 1C). Analysis of the peptide pool by MALDI-TOF and LC–MS/MS indicates that the THP reacts with the amine N-terminus. The absence of THP binding to Cys residues is expected in this case because desIL2 contains only two Cys residues involved in cysteine S–S bond (Table 2).

Similarly to the other protein samples, mass analysis of the reaction product of desIL2 with NODAGA showed the MW of the protein modified with only one chelating agent (Fig. 1D). After digestion of the modified protein, the further analysis of the peptide pool by MALDI-TOF and LC–MS/MS allowed to determine the sequence coverage of desIL2 and peptide identification, as reported in Table 3. The output of the mass analysis showed the identification of peptide 1–7 modified with NODAGA. Results of the mass characterization allow to conclude that the NODAGA chelating agent is introduced only on the N-terminus primary amine of the protein indicating that the primary amine on protein N-terminus reacts in an easier way respect to amine functions present on the side chain of lysine residues within the protein sequence.

**Table 3:** Summary of the mass values identified in the MALDI and LC–MS/MS analysis and assigned to the corresponding peptides in the desIL2 protein

Sequence position	[MH] + (Dalton)	Aminoacid sequence
1–7	707.34	PTSSSTK
1–7	1126.71*	PTSSSTK-***NODAGA***
1–7	1644.77*	PTSSSTK-***THP***
1–8	835.41	PTSSSTKK
38–42	639.35	LTFK
43–47	685.33	FYMPK
48–53	689.32	KKATEL
54–75	2620.40	ELKHLQCLEEELKPLEEVLNLAQSK
76–80	686.32	NFHLR
97–119	2684.23	GSETTFMCEYADETATIVEFLNR
120–132	1496.79	ITFSQSIISTLT

*Sequence of desIL2*

_PTSSSTKKT QLQLEHLLLD LQMILNGINN YKNPKLTRML TFKFYMPKKA TELKHLQCLE EELKPLEEVL NLAQSKNFHL RPRDLISNIN VIVLELKGSE TTFMCEYADE TATIVEFLNR WITFSQSIIS TLT

DesIL2 has no Alanine in position 1 and Cysteine 125 is substituted with a Serine

MH + = protonated monoisotopic mass

### Radiolabelling and quality controls (QCs)

The labelling with Gallium-68 was performed using THP-hrIL2, THP-desIL2 and NODAGA-desIL2. Results of iTLC and HPLC allowed the calculation of LY, RCP and SA (Table 4).

**Table 4:** Labelling yield (LY), radiochemical purity (RCP) and specific activity (SA) of different radiolabelled IL2 molecules

	Labelling yield (LY %)	Radiochemical purity (RCP %)	Specific activity (SA MBq/µg)
[^68^Ga]Ga-THP-hrIL2	58.67 ± 4.81	96.23 ± 1.09	6.02 ± 0.37
[^68^Ga]Ga-THP-desIL2	59.13 ± 2.58	97.91 ± 0.45	6.29 ± 0.52
[^68^Ga]Ga-NODAGA-desIL2	5.47 ± 0.55	96.68 ± 1.86	0.55 ± 0.07

### In-vitro binding to activated T-lymphocytes

#### Phenotypic analysis with fluorescence-activated cell sorter (FACS)

Analysis by flow cytometry showed that after 48 h of culture with PHA-M 45 ± 4% of cells were CD45 + CD25 +. After 72 h of culture, 68 ± 5% of cells were CD45 + CD25 +. For this reason, the culture time of 72 h was selected for binding studies.

#### Kinetic binding assay

**Fig. 2:**
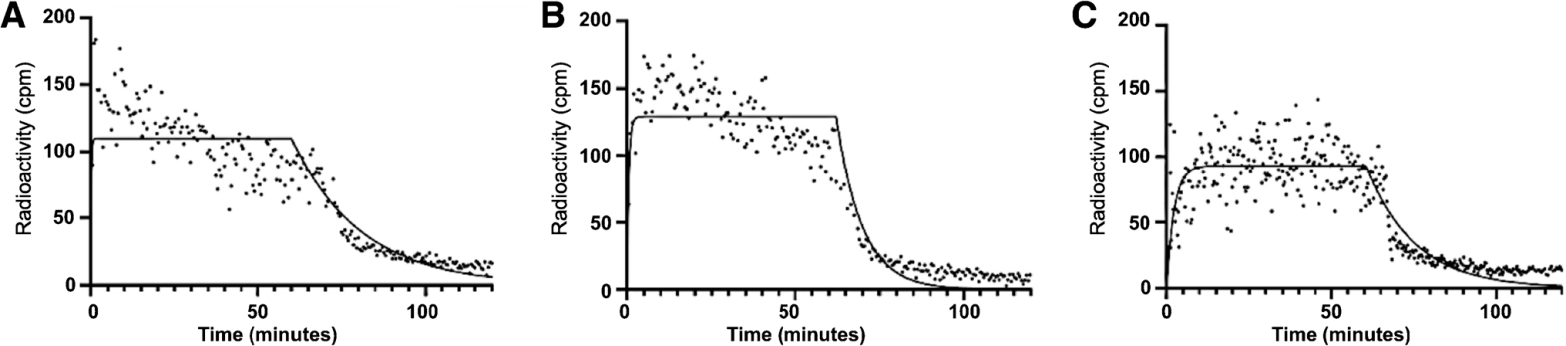
Kinetic binding assay on PHA-activated hPBMC of different radiopharmaceuticals. All radiopharmaceuticals were tested in three different occasions with cells provided by three different donors. **A** [^68^Ga]Ga-THP-hrIL2 in this experiment showed a Kd of 4.33 nM/L. **B** [^68^Ga]Ga-THP-desIL2 in this experiment showed a Kd of 0.863 nM/L. **C** [^68^Ga]Ga-NODAGA-desIL2 in this experiment showed a Kd of 48.7 nM/L

The mean Kd ± standard deviation for [^68^Ga]Ga-THP-hrIL2 was 6.89 ± 2.39 nM/L (Fig. 2A).

[^68^Ga]Ga-THP-desIL2 showed a mean Kd ± standard deviation of 0.584 ± 0.247 nM/L (Fig. 2B).

As control, in order to exclude non-specific binding of the radioligand to BAM molecules present on the surface of Petri-dish, a [^68^Ga]Ga-THP-desIL2 kinetic binding assay was performed on a cell-free BAM-treated Petri-dish showing no significant retention (Supplementary Fig. 1). [^68^Ga]Ga-NODAGA-desIL2 showed a Kd of 67.3 ± 28.1 nM/L (Fig. 2C). Post-hoc analysis: [^68^Ga]Ga-THP-desIL2 vs. [^68^Ga]Ga-THP-hrIL2, *p* = 0.85; [^68^Ga]Ga-THP-desIL2 vs [^68^Ga]Ga-NODAGA-desIL2, *p* = 0.0007; [^68^Ga]Ga-THP-hrIL2 vs [^68^Ga]Ga-NODAGA-desIL2, *p* = 0.001.

### Biodistribution studies in mice

**Fig. 3:**
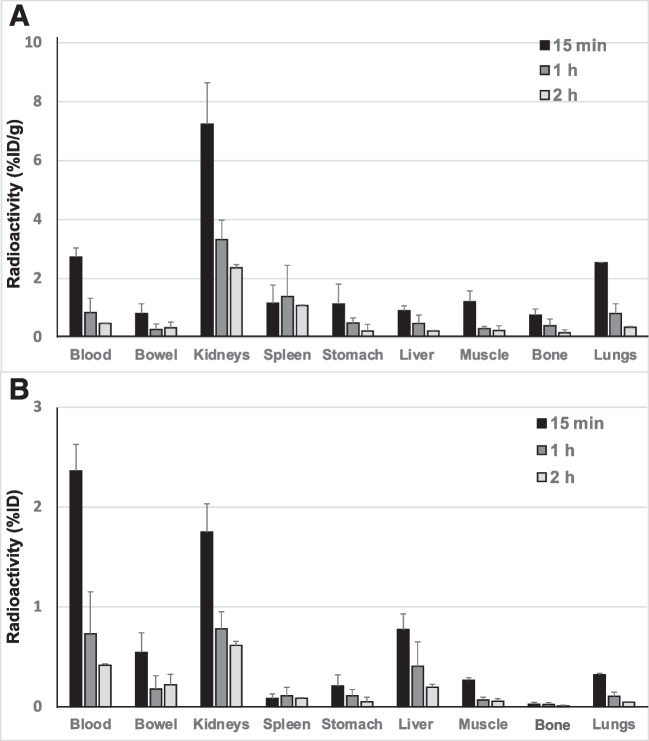
Biodistribution of [^68^Ga]Ga-THP-desIL2 in BALB/c mice at different time points (*n* = 4 per time point). Data are expressed as percentage of injected dose per gram of tissue (%ID/g), (**A**) or as percentage of injected dose per organ (%ID), (**B**). In the lower graph the values of muscle and bone are approximate due to difficulty of evaluating the correct weight of all muscles and bones. Similarly, values observed in bowel can be influenced by the presence of feces. Total blood volume has been calculated as percentage of mouse weight

[^68^Ga]Ga-THP-desIL2 showed an excellent biodistribution in mice, both in terms of %ID/g and %ID with a rapid and predominant kidney metabolism. Little lung uptake was observed at early time points with a quick decrease over time like all other tissues. Spleen uptake showed an increasing uptake at 1 h post-injection (p.i.) (Fig. 3A and B). As a consequence, spleen-to-blood, spleen-to liver and spleen-to muscle ratios increase over time due to the reduction of activity in blood, liver and muscles and the increase of activity in spleen, over time (Table 5 and Supplementary Fig. 2).

**Table 5:** Spleen to Blood ratios in BALB/c mice at different time points after administration of different [^68^Ga]Ga-IL2 formulations

	15 min Mean ± SD (95% CI)	1 h Mean ± SD (95% CI)	2 h Mean ± SD (95% CI)	*p*
[^68^Ga]Ga-THP-desIL2	0.44 ± 0.28 (-0.26 to 1.14)	1.78 ± 0.58 (0.34 to 3.22)	2.80 ± 0.22 (2.25 to 3.34)	0.001
[^68^Ga]Ga-THP-hrIL2	0.45 ± 0.15 (0.07 to 0.83)	0.60 ± 0.23 (0.02 to 1.18)	1.10 ± 0.16 (0.70 to 1.50)	0.01
[^68^Ga]Ga -NODAGA-desIL2	0.48 ± 0.23 (0.12 to 0.85)	0.35 ± 0.22 (-0.21 to 0.90)	0.23 ± 0.11 (-0.05 to 0.52)	0.32

[^68^Ga]Ga-THP-desIL2: 15 min *vs*. 1 h, *p* = 0.006; 15 min *vs*. 2 h, *p* = 0.0003; 1 h *vs*. 2 h, *p* = 0.02

[^68^Ga]Ga-THP-hrIL2: 15 min *vs*. 1 h, *p* = 0.36; 15 min *vs*. 2 h, *p* = 0.005; 1 h *vs*. 2 h, *p* = 0.01

*SD*, Standard Deviation

*CI*, Confidence Interval

[^68^Ga]Ga-THP-hrIL2 showed rapid kidney excretion but an unexpected high lung uptake at 15 min p.i. and low spleen uptake which decreased approximately by 50% each hour. [^68^Ga]Ga-NODAGA-desIL2 showed a very fast blood clearance but high liver uptake and low spleen uptake. Therefore, by using these last two formulations, we observed a decreased Spleen/Bkg ratio over time.

### Development of a ready-to-use lyophilized kit for labelling with Gallim-68

THP-desIL2 stored at -80 °C showed high stability for up to 6 months. We observed a small, non significant, reduction of LY and SA with time but unmodified RCP, as shown in Table 6.

**Table 6:** Labelling yield (LY), Specific Activity (SA) and Radiochemical Purity (RCP) of [^68^Ga]Ga-THP-desIL2 after storage at -80 °C

	Labelling yield (LY %)	Radiochemical purity (RCP %)	Specific activity (SA MBq/µg)
Freshly prepared	58.67 ± 4.81	97.9 ± 0.4	6.3 ± 0.5
After 1 month	58.18 ± 0.85	97.4 ± 0.6	6.1 ± 0.5
After 2 months	57.29 ± 2.69	96.9 ± 1.3	6.1 ± 0.7
After 3 months	57.15 ± 1.57	96.2 ± 1.1	6.0 ± 0.4
After 6 months	51.80 ± 3.74*	96.5 ± 2.7	5.6 ± 0.6

*LY of freshly prepared [^68^Ga]Ga-THP-desIL2 *vs.* 6 months, *p* = 0.008

iTLC analysis confirmed that THP-desIL2, when stored at -80 °C, can be radiolabelled with Gallium-68 with high RCP. Results of SDS-PAGE showed that THP-desIL2 stored at -80 °C may form dimeric complexes after 3- and 6-months storage (Supplementary Fig. 3) thus explaining the low SA.

Stability of [^68^Ga]Ga-THP-desIL2 in human serum at 37 °C, evaluated by iTLC, was 98.5 ± 1.9% of basal value after 1 h, 97.6 ± 2.1% after 2 h, 90.7 ± 1.5 after 3 h (*p* < 0.0001 vs. basal value) and 85.9 ± 2.1 after 4 h (*p* < 0.0001 vs. basal value).

### Biodistribution and dosimetry of [^68^Ga]Ga-THP-desIL2 in humans

**Fig. 4:**
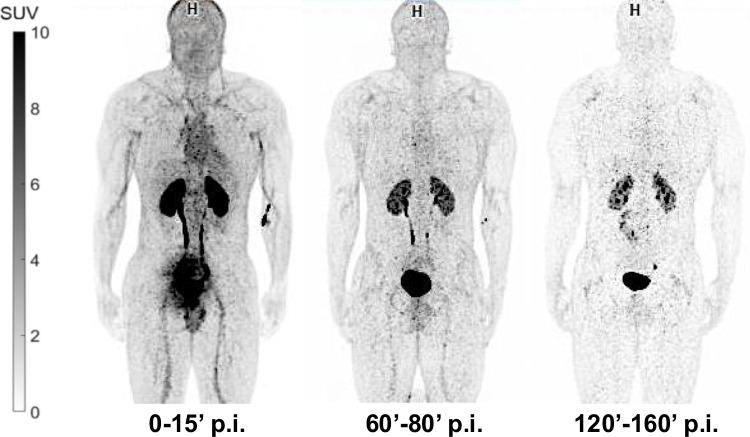
Biodistribution of [^68^Ga]Ga-THP-desIL2 in a normal subject at different time points post injection. High vascular uptake can be seen at 15 min, decreasing with time. A quick kidney metabolism can be detected

Human studies confirmed the biodistribution observed in mice with a quick and predominant kidney metabolism, high vascular uptake in the first minutes after the injection of the radiopharmaceutical, which normalizes at 1 h p.i. (Fig. 4).

None of the patients experienced any side effect during or after the injection of [^68^Ga]Ga-THP-desIL2. Blood counts at different time points after tracer administration did not show any significant modification of blood cell subsets (Supplementary Table 1).

Effective dose (ICRP 103) was 0.0139 mSv/MBq (Supplementary Table 2). A diagnostic PET scan had an effective dose of 2.21 mSv (without CT). As expected from pre-clinical studies and from biodistribution, the greatest absorbed organ dose was found in the urinary bladder (0.0639 mSv/MBq) followed by the kidneys (0.0225 mSv/MBq), spleen (0.0081 mSv/MBq), heart (0.0069 mSv/MBq), liver (0.0054 mSv/MBq) and lymph nodes (0.0044 mSv/MBq).

## Discussion

In the past three decades, radiolabelled IL2 has demonstrated its potential for imaging activated T-lymphocytes in many autoimmune diseases and in different cancers. Several formulations of radiolabelled-IL2 have been investigated in both preclinical and clinical setting by us and by others [13–31, 34]. However, each radiopharmaceutical had some limitation, either high cost, long synthesis time, poor labelling efficiency, non-physiological distribution or high liver uptake.

Among the recently developed PET radiopharmaceuticals, [^18^F]-FB-IL2 requires several synthesis modules and the production is expensive and time consuming, [^18^F]-AlF-RESCA-IL2 showed the highest in vitro uptake in target tissues in mice but with prevalent hepatobiliary clearance, [^68^Ga]Ga-NODAGA-IL2 showed low production yield [28]. All the above-mentioned radiopharmaceuticals used desIL2 as protein and hrIL2 has never been tested with positron-emitters, although gave excellent results when labelled with Iodine-123. Van der Veen et al., in particular, tested in vitro and in vivo the same radiopharmaceutical we also used in our study [^68^Ga]Ga-NODAGA-desIL2, although the synthesis process was slightly different from our and no information has been published on the biochemical characterization.

Since both THP and NODAGA are stable chelating agents at room temperature, we radiolabelled desIL2 and hrIL2 with both THP or NODAGA and performed several in-vitro and in-vivo experiments to fully characterize and compare these radiopharmaceuticals for PET use.

The four resulting formulations (THP-desIL2, THP-hrIL2, NODAGA-desIL2 and NODAGA-hrIL2), analyzed with mass spectrometry, showed that 79% of desIL2 was conjugated with 1 THP molecules, 59% of hrIL2 was conjugated with 1 molecule of THP, only 5% of desIL2 was conjugated with a molecule of NODAGA whereas no hrIL2 was conjugated with NODAGA (0.6%). Based on these data, NODAGA-hrIL2 was excluded from further investigation, whereas the other formulations underwent further in-vitro and in-vivo experiments.

Kinetic binding assay performed on PHA-activated hPBMC, expressing IL2R α-chain, showed that the radiolabelled IL2 is able to bind to its specific receptor within 10 min maintaining high affinity. In particular, THP-desIL2 showed the highest Kd (5.84 × 10^–10^ ± 2.47 × 10^–10^ mol/L), compared to the other formulations.

Furthermore, [^68^Ga]Ga-THP-desIL2 showed the best biodistribution in mice with the highest %ID/g in spleen at 1 h and 2 h p.i., as expected for the physiological presence of activated T-cells, and with increasing Spleen/Bkg ratios over time. This was not observed using the other two formulations. Liver uptake was also lower with [^68^Ga]Ga-THP-desIL2, that showed a predominant renal metabolism as described for unlabelled IL2 [35].

Moving to human application, we first developed and patented a ready-to-use kit containing lyophilized THP-desIL2 for easy labelling with Gallium-68 at room temperature. The stability tests performed to assess the shelf life of THP-desIL2, demonstrated that the kit remains stable if stored at −80 °C up to 6 months maintaining high RCP and a small reduction of LY and SA over time. Indeed, after 3 and 6 months, THP-desIL2 may form some dimeric complexes. Although, we believe that these dimeric complexes can be dissolved by an appropriate dilution during the radiolabelling, since no other forms but the monomeric one, can be observed by HPLC analysis after radiolabelling, dimerization of IL2 in the lyophilized kit could be the reason for the lower LY and the small reduction in SA. However, SA remains quite stable and within acceptable limits for diagnostic purposes, which is important for the product's stability over time.

[^68^Ga]Ga-THP-desIL2, used for first-in-human PET/CT studies, demonstrated excellent biodistribution with predominant kidneys metabolism, favorable dosimetry and most important, no adverse effects were observed thus confirming that the administration of IL2 at sub-pharmacological dose is safe. Since this phase I study was crucial to assess safety, dosimetry and biodistribution of [^68^Ga]Ga-THP-desIL2 in human subjects, it was conducted in only 5 healthy volunteers. Larger phase II studies are needed to assess the role of this innovative imaging strategy for diagnosis and therapy follow-up in different clinical applications.

If these encouraging preliminary results will be further confirmed in larger patient population, this approach would potentially become a unique opportunity to non-invasively monitor in vivo CD25 + T-cell trafficking in a wide spectrum of diseases, with particular regard to autoimmune diseases and cancer.

Several other alternative approaches have been proposed to image different immune cells population or pathways. Amongst them, [^18^F]F-AraG, that mainly detects activated CD8 + cells [36–39], ^89^Zr- or ^64^Cu-anti-CD8 diabodies [40–42], that can target both activated and resting CD8 cells, ^64^Cu-anti-CD4 diabodies [43], that can target CD4 + cells, are all showing promising perspectives. However, radiolabelled IL2 has the advantage to show the highest specificity since targets a single cell population, namely CD25 + T-cells, involved in the pathogenesis of autoimmune disease and in cancer progression. Further phase II-III studies are warranted to identify the role of each of these compounds for diagnosis, prognosis or therapy follow-up. Overall, these new radiopharmaceuticals offer the possibility to investigate the physiology and pathophysiology of lymphocyte subsets, and their clinical relevance is currently being investigated.

## Conclusions

We set-up an efficient and reproducible method to radiolabel IL2 in GMP conditions, with Gallium-68 at room temperature, without loss of receptor binding activity. This method includes the use of a bifunctional chelator (THP) efficiently conjugated with IL2, lyophilized in a ready-to use kit that can be stored at −80 °C for at least 6 months. Results in mice and humans showed excellent biodistribution, safety and a favorable dosimetry. [^68^Ga]Ga-THP-IL2 in clinical practice can be an important tool for in vivo tracking of activated T-cells.

## Supplementary Information

Below is the link to the electronic supplementary material.

Supplementary file1 (PDF 41 KB)

Supplementary file2 (PDF 13 KB)

Supplementary file3 (PDF 221 KB)

Supplementary Tables 1 and 2 (DOCX 17 KB)

## Data Availability

All experimental data are available upon request to the corresponding author.
