# Supplementary figures and images for: [68Ga]Ga-interleukin-2 for imaging activated T-lymphocytes: biochemical characterization and phase I study in normal subjects
Source: Eur J Nucl Med Mol Imaging. 2025 Jul 1;53(1):544–56. doi: 10.1007/s00259-025-07430-9 (PMC12660392; doi:10.1007/s00259-025-07430-9)

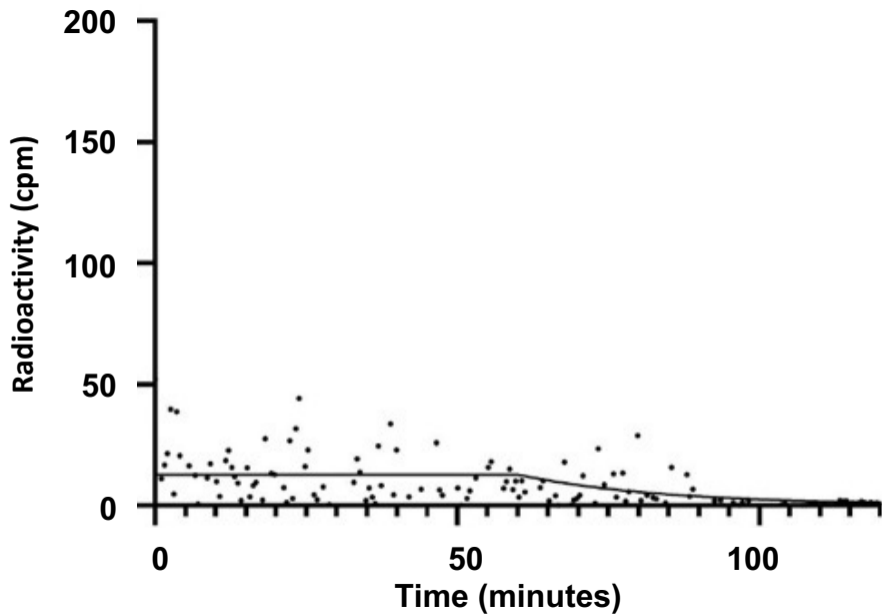

Supplement: Supplementary file 1 — Supplementary file1 (PDF 41 KB) [file 259_2025_7430_MOESM1_ESM.pdf]

Spleen/Blood activity ratio  
(%ID/g)

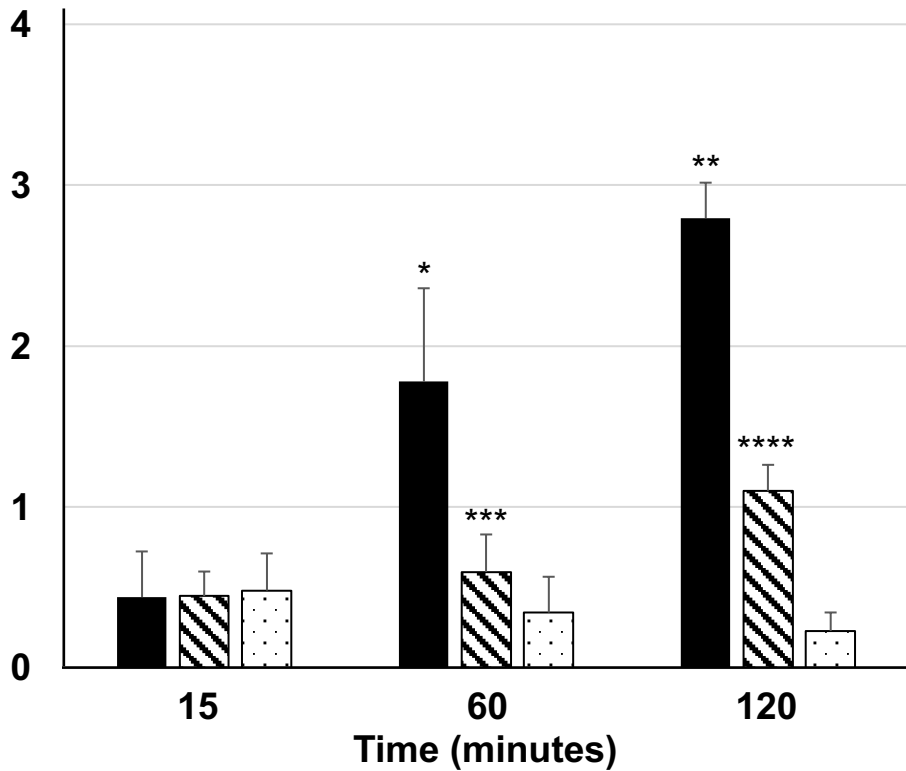

Supplement: Supplementary file 2 — Supplementary file2 (PDF 13 KB) [file 259_2025_7430_MOESM2_ESM.pdf]

Markers

1 month

3 months

6 months

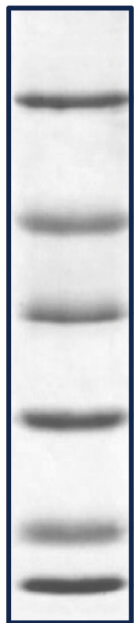

— 66.2 kDa

— 45.0 kDa

— 35.0 kDa

— 25.0 kDa

— 18.4 kDa

— 14.4 kDa

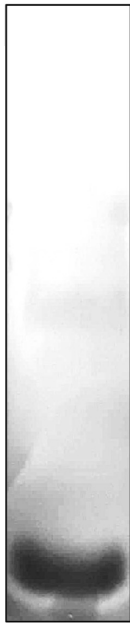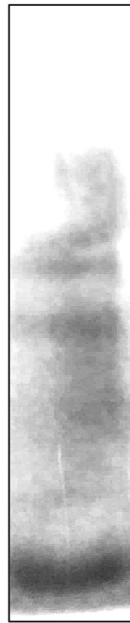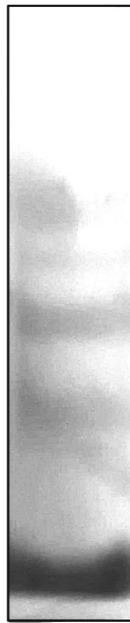

Supplement: Supplementary file 3 — Supplementary file3 (PDF 221 KB) [file 259_2025_7430_MOESM3_ESM.pdf]
