# Supplementary material for: [68Ga]Ga-interleukin-2 for imaging activated T-lymphocytes: biochemical characterization and phase I study in normal subjects
Source: Eur J Nucl Med Mol Imaging. 2025 Jul 1;53(1):544–56. doi: 10.1007/s00259-025-07430-9 (PMC12660392; doi:10.1007/s00259-025-07430-9)
Supplement: Supplementary file 4 — Supplementary Tables 1 and 2 (DOCX 17 KB) [file 259_2025_7430_MOESM4_ESM.docx]

Supplementary Table 1

Results of blood count at different time points after 68Ga-THP-desIL2 administration. Data are mean±SD of 5 normal subjects (all comparisons are not statistically significant).

|  | **Before injection** | **60 min p.i.** | **120 min p.i.** |
| --- | --- | --- | --- |
| **WBC (*1000/µL)** | 6.56±2.14 | 6.40±2.61 | 6.35±2.28 |
| **NEU (*1000/µL)** | 3.82±1.71 | 3.82±2.13 | 3.81±2.05 |
| **LYM (*1000/µL)** | 1.98±0.26 | 1,86±0.20 | 1.86±0.08 |
| **MONO (*1000/µL)** | 0.51±0.25 | 0,49±0.28 | 0,45±0.26 |
| **EOS (*1000/µL)** | 0.21±0.10 | 0,19±0.06 | 0.18±0.08 |
| **BASO (*1000/µL)** | 0.01±0.02 | 0.01±0.02 | 0.01±0.02 |
| **RBC (*1000000/µL)** | 4.91±0.47 | 4.83±0.76 | 4.97±0.55 |
| **Hb (g/dL)** | 14.95±1.17 | 14.78±1.98 | 15.11±1.39 |
| **HCT (%)** | 44.92±3.63 | 44.10±6.25 | 45.50±4.24 |
| **MCV (fL)** | 91.77±1.69 | 91.62±1.78 | 91.72±1.83 |
| **MCH (pg)** | 30.62±1.00 | 30.75±1.26 | 30.57±1.12 |
| **MCHC (%)** | 33.35±0.70 | 33.52±1.00 | 33.30±0.63 |
| **RDW (%)** | 11.92±5.6 | 12.00±6.7 | 11.90±0.60 |
| **PLT (*10000/µL)** | 23.20±6.33 | 23.50±7.46 | 22.85±6.39 |
| **MPV (µm)** | 7.65±1.45 | 7.59±1.38 | 7.79±1.19 |

Supplementary Table 2

Results of effective dose calculation

by IDAC-Dose2.1 software

| **Organs [mGy/MBq]** | **Male** |
| --- | --- |
| Adrenals | 4.89e-03 |
| Brain | 7.04e-04 |
| Breast | 1.68e-03 |
| Colon wall | 1.98e-03 |
| ET region | 4.20e-04 |
| Gallbladder wall | 2.50-03 |
| Heart wall | 6.92e-03 |
| Kidneys | 2.25e-02 |
| Liver | 5.41e-03 |
| Lung | 6.35e-02 |
| Lymphatic nodes | 4.47e-03 |
| Muscle | 9.63e-04 |
| Pancreas | 1.85e-03 |
| Bone marrow | 2.61 e-03 |
| Salivary glands | 3.91e-04 |
| Small intestine wall | 2.38e-03 |
| Spleen | 8.10e-03 |
| Stomach wall | 2.30e-03 |
| Testes | 6.30e-04 |
| Thyroid | 2.01e-03 |
| Urinary bladder wall | 6.39e-02 |
| **Effective dose 103 [mSv/MBq]** | **1.39e-02** |
